# Endoreplication Controls Cell Fate Maintenance

**DOI:** 10.1371/journal.pgen.1000996

**Published:** 2010-06-24

**Authors:** Jonathan Bramsiepe, Katja Wester, Christina Weinl, Farshad Roodbarkelari, Remmy Kasili, John C. Larkin, Martin Hülskamp, Arp Schnittger

**Affiliations:** 1 Institut de Biologie Moléculaire des Plantes du CNRS, Université de Strasbourg, Strasbourg, France; 2 Lehrstuhl für Botanik III, Universität zu Köln, Köln, Germany; 3 Unigruppe am Max-Planck-Institut für Pflanzenzüchtungsforschung, Lehrstuhl für Botanik III, Universität zu Köln, Köln, Germany; 4 Department of Biological Sciences, Louisiana State University, Baton Rouge, Louisiana, United States of America; Peking University, China

## Abstract

Cell-fate specification is typically thought to precede and determine cell-cycle regulation during differentiation. Here we show that endoreplication, also known as endoreduplication, a specialized cell-cycle variant often associated with cell differentiation but also frequently occurring in malignant cells, plays a role in maintaining cell fate. For our study we have used Arabidopsis trichomes as a model system and have manipulated endoreplication levels via mutants of cell-cycle regulators and overexpression of cell-cycle inhibitors under a trichome-specific promoter. Strikingly, a reduction of endoreplication resulted in reduced trichome numbers and caused trichomes to lose their identity. Live observations of young Arabidopsis leaves revealed that dedifferentiating trichomes re-entered mitosis and were re-integrated into the epidermal pavement-cell layer, acquiring the typical characteristics of the surrounding epidermal cells. Conversely, when we promoted endoreplication in glabrous patterning mutants, trichome fate could be restored, demonstrating that endoreplication is an important determinant of cell identity. Our data lead to a new model of cell-fate control and tissue integrity during development by revealing a cell-fate quality control system at the tissue level.

## Author Summary

Differentiating cells often amplify their nuclear DNA content through a special cell-cycle variant, called endoreplication, in which cell division is skipped. Although this process is widespread from humans to plants, not much is currently known about the biological importance of endoreplication. Moreover, the control of cell-cycle activities has been thought to follow developmental decisions and the adoption of a specific cell fate. Here we have uncovered a previously unrecognized function of endoreplication in maintaining cell identity, presenting a striking example of how cell fate and cell-cycle progression are linked. Using leaf hairs on the reference plant Arabidopsis as a model, we show that compromising endoreplication leads to dedifferentiation of the newly forming leaf hair cell. Live observations of young Arabidopsis leaves revealed that dedifferentiating leaf hairs underwent repeated rounds of cell division and were re-integrated into the epidermal cell layer acquiring the typical characteristics of the surrounding epidermal cells. Conversely, promoting endoreplication in mutants that fail to develop hairs could at least partially restore their differentiation program. With this, our findings also pinpoint an important role of the social context of a cell, revealing a differentiation control system at the tissue level.

## Introduction

Many different cell cycle programs can be found in developing multicellular organisms [1]. Typically, embryonic cell cycles are short with a rapid sequence of the DNA-synthesis phase (S-phase) and mitosis (M-phase) rapidly generating cells or nuclei in a syncytium. At later stages of development, differentiating cells in many animal and plant species frequently enter an endoreplication cycle in which mitosis is skipped and DNA is re-replicated leading to polyploid cells [2]–[4]. However, very little is known about the biological importance of endoreplication and the resulting cellular polyploidy.

Progression of mitotic cell cycles is controlled by cyclin-dependent kinase (CDK)–cyclin heterodimeric complexes. Their action is in particular required for the entry into S-phase and M-phase [5]. The major determinant of CDK activity is the abundance of the cyclin co-factor and cyclin levels are controlled both at the level of transcription and by protein degradation [6]. In particular two multi-protein complexes, the Skip-F-box-Cullin (SCF) complex and the Anaphase-Promoting-Complex/Cyclosome (APC/C) ligate ubiquitin moieties to cyclins marking them for subsequent degradation by the proteasome [7], [8]. The specificity of these ubiquitin ligases is brought about by adaptor proteins, i.e. F-box proteins for the SCF complex and Cdh1/FZR/CCS52 and Cdc20/FZY for the APC/C [8]–[12].

In addition to cyclins, CDKs are also controlled by the binding of inhibitors, for instance p27^Kip1^ in mammalian cells [13]. Moreover, CDK activity is regulated by posttranslational modifications such that phosphorylation of a conserved threonin residue (position 161 in Arabidopsis) in the so-called T-loop is absolutely required for kinase activity [5], [14], [15]. Conversely, phosphorylation of two residues in the P-loop (typically T14 and Y15) can block kinase activity. All these control mechanisms appear to be globally conserved in eukaryotes and are present from yeast to plants [16]–[18].

Similarly to the different types of cell division cycles that exist, there are also many different endocycle variants [1], [2]. However, it appears that endoreplication and mitotic cycles share many of the important regulators, and likely S-phase cyclins and CDKs are also key components of an endoreplication cycle [2]. A well-studied example of endoreplicating plant cells are Arabidopsis leaf hairs (trichomes) [19], [20]. Trichomes are single epidermal cells that develop on almost all aerial structures of Arabidopsis. They are regularly spaced and it has been found that patterning relies on a substrate-depletion and lateral inhibition mechanism [21]–[23]. The trichome pattern is established in the basal part of the leaf where the positive regulators (activators of trichome fate) GLABRA1 (GL1), a R2R3 MYB transcription factor, GLABRA3 (GL3), a bHLH transcription factor, and TRANSPARENT TESTA GLABRA1 (TTG1), WD-40 protein, are initially ubiquitously expressed.

The current model postulates that due to stochastic fluctuations some cells express these regulators at a higher concentration and these differences in expression levels become greatly enhanced due to a positive feed back loop of the activator complex. In turn, the positive regulators induce the expression of inhibitors, small R3 single repeat MYB transcriptional regulators that are then released from cells that have high levels of activators and inhibit the formation of activator complexes in the surrounding cells [21], [23]. So far six inhibitors have been identified: TRIPTYCHON (TRY), CAPRICE (CPC), ENHANCER OF TRY AND CPC (ETC) 1, 2, 3 (also called CPC-LIKE PROTEIN [CPL]), and TRICHOMELESS (TCL) [24]–[28]. The current model suggests that once a cell has reached a certain threshold of activator complex, trichome fate is established and the incipient trichome cell starts to express downstream genes, such as the HD bZIP transcription factor GLABRA2 (GL2). Many downstream trichome-specific genes are then activated that regulate further outgrowth of the formation of typically three to four branches [29]–[31].

One of the earliest signs of trichome differentiation is the entry into an endoreplication cycle and along with further outgrowth trichomes undergo usually three to four rounds of DNA replication leading to a final DNA content of approximately 32C [19], [20], [32]. Endoreplication correlates well with trichome growth and typically mutants that have reduced endoreplication levels also display smaller trichomes with fewer branches while mutants with increased endoreplication levels have larger trichomes with more branches [33], [34].

Two core cell-cycle regulators have so far been shown to be required for the trichome endoreplication cycle. The first one is CDKA;1, the major regulator of mitotic cycles and the Arabidopsis homolog of the yeast Cdc2/CDC28 kinase. Either a substitution of the CDKA;1 amino acid T161 with D161 (further on abbreviated as *D*) or a mutant in which T14 has been substituted by D14 and Y15 by E15 (abbreviated as *DE*) resulted in reduced CDKA;1 kinase activity and both weak loss-of-function alleles displayed smaller nuclei in trichomes along with a reduction of trichome size [14], [17].

The other cell-cycle regulator involved in trichome cell-cycle control is SIAMESE (SIM), a putative CDK inhibitor, and in *sim* mutants endoreplication is partially converted into a mitotic program resulting in multicellular trichomes [35], [36]. One likely target of SIM is a CDK-cyclin D complex since a paralog of SIM in rice can inhibit cyclin D action when expressed in yeast [37]. In addition, the misexpression of CYCLIN D3;1 (CYCD3;1) in trichomes has resulted in the formation of multicellular trichomes [38]. A second target of SIM could be mitotic B-type cyclins since also the ectopic expression of a constitutive active B-type cyclin resulted in multicellular trichomes [39], [40].

Remarkably, some of the trichome patterning genes appear to have a function in endoreplication control. Loss of GL3 function results in a reduction of endoreplication levels and conversely, *try* mutants undergo one additional round of endoreplication [34]. In addition, GL1 might also control endoreplication although the situation is less clear since in one report the overexpression of *GL1* was found to result in increased endoreplication levels in trichomes while in another study no such effect in was observed [41], [42]. Interestingly, it has been found that *SIM* is a direct early target of GL3 and GL1 pinpointing to a tight interaction between patterning genes and the regulation of the endoreplication cycle [43].

Here we have dissected the relationship between pattern formation and cell-cycle control by using plants with reduced endoreplication levels in trichomes. We found that trichome fate is surprisingly plastic and trichome fate can be changed into an epidermal pavement cell fate even in advanced stages of trichome differentiation. Our data show that progression through an endoreplication cycle is an important aspect of cell fate acquisition and is crucial for cell fate maintenance.

## Results

### Reduction of endoreplication is correlated with a decrease in trichome numbers

**Figure 1:**
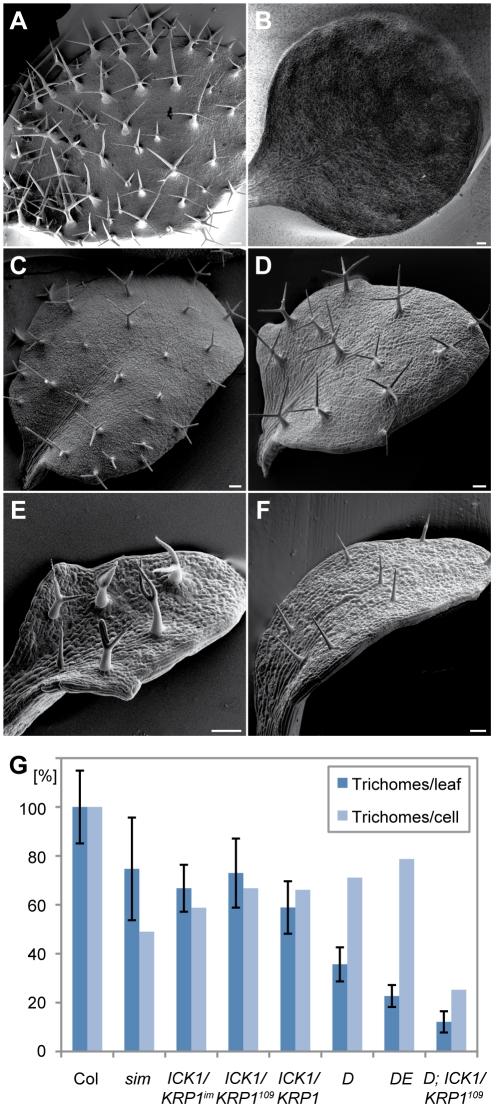
Reduced trichome numbers on plants with altered cell-cycle control in trichomes. Scanning electron micrographs of rosette leaf number 4 of Arabidopsis seedlings. (A) Columbia, (B) *PRO_CPC_:CYCD3;1*, (C) *PRO_GL2_: ICK1/KRP1^im^*, (D) *CDKA;1^T161D^*, (E) *CDKA;1^T14D/Y15E^* and (F) *PRO_GL2_:KRP1^109^* - *CDKA;1^T161D^* plants. (G) Quantification of trichome numbers of leaf 3 and 4 in comparison with Columbia. Col Columbia n = 31. *sim siamese* n = 18. *ICK1/KRP1^IM^* ^,^ *PRO_GL2_:GUS:YFP:KRP1^109^* n = 30. *KRP1^109^ PRO_GL2_:KRP1^109^* n = 32. *D CDKA;1^T161D^* n = 32. *DE CDKA;1^T14D/Y15E^* n = 32. *D; ICK1/KRP1^109^ CDKA;1^T161D^; PRO_GL2_:KRP1^109^* n = 26. Error bars: standard deviation. Scale bars: (A–F) 100 µm.

Since several trichome patterning mutants are also affected in endoreplication control, we asked whether there is a functional connection between cell-cycle progression and pattern formation during very early trichome development. We therefore first revisited *sim* mutants in which trichomes undergo cell divisions leading to multicellular trichome [35], [36]. Remarkably, we found that under our growth conditions *sim* mutants develop significantly fewer (T-test with p = 0.0001) trichome initiates sites (TIS) per leaf in comparison to wild type (Figure 1G, dark blue bars). A patterning defect of *sim* mutants became even more prominent when we compared the number of trichomes per epidermal cell number (light blue bars), here *sim* mutants had only approximately half of the trichomes found in wild type.

To test whether cell-cycle progression and trichome initiation are functionally linked, we sought for additional possibilities to promote cell proliferation in trichomes. In earlier experiments, we have used the *GL2* promotor to drive expression of *CYCD3;1* or a N-terminally truncated B-type cyclin resulting in the formation of multicellular trichomes. Both the multicellular trichomes and the remaining single-celled trichomes, displayed a strong reduction in their ploidy level indicating that a mitotic cycle could inhibit and override an endoreplication cycle [38]–[40].

To manipulate cells during the initial patterning process we used here the promoters of the *CPC* and *TRY* gene that are active earlier than the *GL2* promoter to drive expression of *CYCD3;1* [44]. Similar to the expression of *CYCD3;1* from the *GL2* promoter, expression from the *CPC* and the *TRY* promoter resulted in multicellular trichomes. However, in addition, we obtained more than eight transgenic lines that were devoid of trichomes out of more than 20 primary transformants (Figure 1A and 1B).

We envisioned two scenarios that could explain these results. First, expression of *CDCD3;1* and the loss of the CDK inhibitor SIM might interfere with trichome-fate establishment by promoting/allowing cell division of an incipient trichome cell and, thereby constantly diluting the presumptive activator levels. Alternatively, or in addition, endoreplication cycles may be instrumental for trichome patterning. To discriminate between these two possibilities, we analyzed plants that have reduced endoreplication levels in trichomes.

Previously, we had generated plants that express the CDK inhibitor *ICK1/KRP1* under the trichome-specific *GL2* promoter, which led to strongly reduced endoreplication levels in trichomes [45]. This effect was even stronger when the N-terminally truncated ICK1/KRP1 variant ICK1/KRP1^109–191^, which lacks the first 108 amino acids was expressed from the GL2 promoter. The ICK1/KRP1^109–191^ form of the protein displays increased protein stability and interacts more strongly with CDKA;1 and CYCD3;1 than the full length ICK1/KRP1 protein in yeast two hybrid experiments [45]–[49]. Examination of trichome numbers on leaves of these *ICK1/KRP1*-misexpressing plants revealed that trichome numbers were reduced (Figure 1G and Table S1). To complement this set of experiments, we analyzed two weak *cdka;1* mutants, *D* and *DE*, that were previously found to display reduced endoreplication levels in leaves [14], [17]. None of these genotypes resulted in multicellular trichomes or would be expected to favor increased cell division, making dilution of cell fate transcriptional activators unlikely as an explanation of the observed reduction in trichome number.

**Figure 2:**
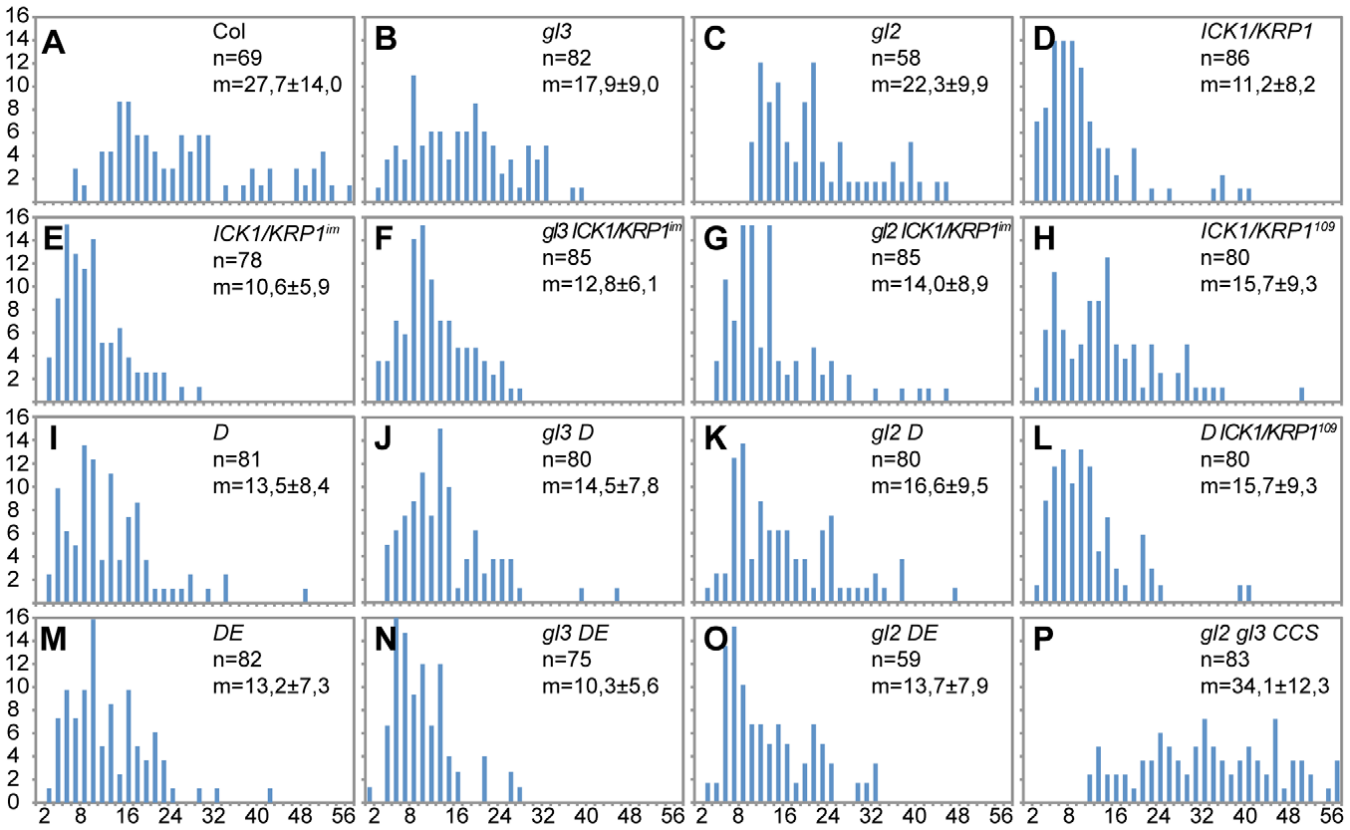
Analysis of DNA content in trichomes. (A–P) Distribution of the trichome DNA contents in relative fluorescence units (RFU). Two RFUs roughly represent 2C, calibrated with wild-type and *gl3* trichome nuclei. (A) Wild type. (B) *glabra3* (*gl3*). (C) *glabra2* (*gl2*). (D) *PRO_GL2_:ICK1/KRP1*. (E) *PRO_GL2_:GUS:YFP:ICK1/KRP1^109-191^* (*ICK1/KRP1^im^*). (F) *gl3 - ICK1/KRP1^im^*. (G*) gl2 - ICK1/KRP1^im^*. (H) *PRO_GL2_:ICK1/KRP1^109–191^*. (I) *CDKA;1^T161D^* (*D*). (J) *gl3 - D*. (K) *gl2 - D*. (L) *D* - *PRO_GL2_:ICK1/KRP1^109–191^*. (M) *CDKA;1^T14D/Y15E^* (*DE*). (N) *gl3* - *DE*. (O) *gl2* - *DE*. (P) *gl2* - *gl3* - *PRO_GL2_:CCS52A1*.

Quantification of DAPI-stained trichomes revealed that both D and DE plants have decreased endoreplication levels in trichomes in comparison with wild-type plants (Figure 2I and 2M) and consistent with the above obtained results with ICK1/KRP1-misexpressing plants both weak *cdka;1* loss-of-function mutants also displayed fewer trichomes on leaves than control plants (Figure 1D, 1E, 1G, and Table S1). To reduce endoreplication levels further, we combined D plants with plants misexpressing ICK1/KRP1^109–191^. These plants displayed the severest trichome effect among the cell-cycle mutants studied and total trichome numbers dropped almost ten fold in comparison to wild type (Figure 1F and 1G and Table S1).

It was previously shown that ICK1/KRP1 can act non-cell-autonomously and thus, *PRO_GL2_:ICK1/KRP1*-expressing plants have typically fewer but larger epidermal cells that surround trichomes [46]. Plants with a reduction of CDK activity either due to a strong overexpression of CDK inhibitors or due to a compromised kinase, as in *D* and *DE* plants, have also generally larger epidermal cells [14], [17], [50], [51]. This increase in cell size makes it difficult to discriminate between a reduction of trichome number due to reduced leaf size and fewer cells versus a *bona fide* patterning defect. To correct for cell-size and cell-number differences, we determined the ratio of trichomes per epidermal cells. This estimate revealed that there is indeed a true trichome patterning defect in both *ICK1/KRP1* overexpressing plants as well as in *D* and *DE* plants (Figure 1G, light blue bars; Table S1).

To substantiate that the reduction of trichome number is due to a true patterning defect rather than due to alterations of leaf growth, we analyzed plants expressing a cell-autonomous version of ICK1/KRP1 in trichomes; in this construct, the N-terminally truncated CDK inhibitor ICK1/KRP1^109–191^ is fused to GUS and GFP [46], [49]. We could also observe significantly reduced trichome numbers in plants expressing the immobile GUS:YFP:ICK1/KRP1^109–191^ (hereafter referred to as ICK1/KRP1^im^) under the *GL2* promoter (T-test, p = 0.0001) confirming that local interference with endoreplication results in a patterning defect proper (Figure 1C and 1G and Table S1).

### Genetic interactions between plants with reduced endoreplication levels and patterning mutants

**Figure 3:**
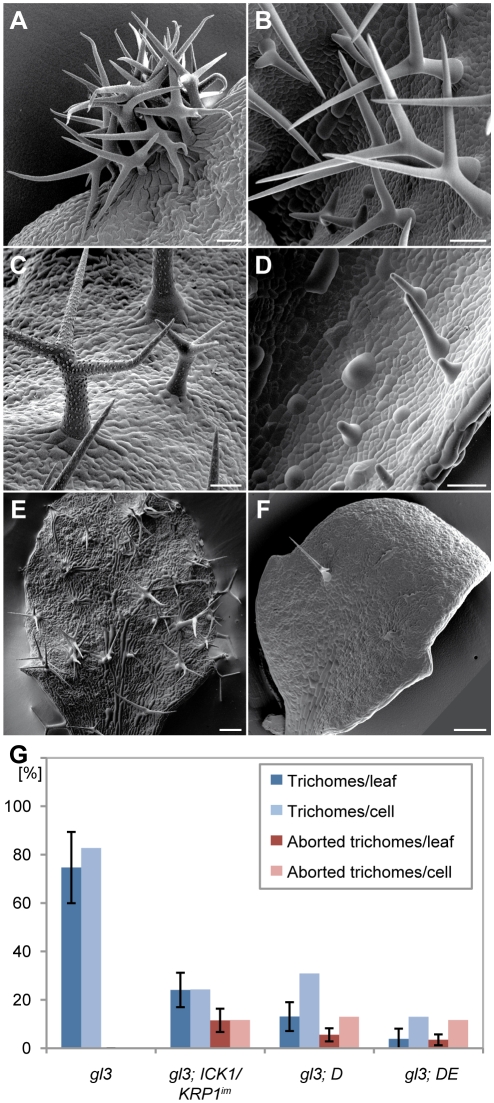
Genetic combinations of trichome patterning mutants with plants that have a reduced endoreplication levels in trichomes. Scanning electron micrographs of rosette leaf number 4. (A) *cpc try*. (B) *gl3*. (C) *PROGL2:KRP1^109^* in *cpc try*. (D) *PROGL2:KRP1^109^* in *gl3*. (E) *CDKA;1T^161D^* in *cpc try* (F) *CDKA;1T^161D^* in *gl3*. (G) Quantification of trichome numbers of leaf 3 and 4 in comparison with wild type (Columbia). Error bars: standard deviation. Scale bars: (A–D) 50 µm; (E,F) 200 µm.

To dissect the epistasis of the relationship between endoreplication and pattern formation, we introgressed the weak *cdka;1* loss-of-function mutant *D* and *DE* as well as *ICK1/KRP1*-overexpressing plants into several trichome patterning mutants. First, we analyzed the effect of reduced endoreplication in *cpc-try* double mutants that develop large trichome clusters due to reduced lateral inhibition [44] (Figure 3A). The combination with plants expressing the *PRO_GL2_:ICK1/KRP1^109–191^* construct or *D* and *DE* plants strongly reduced cluster formation and cluster size (Figure 3C and 3E): 56 percent of trichome initiation sites now contain only one trichome and approximately 2/3 of the clusters form just two trichomes whereas in *cpc-try* double mutant 95 percent of the sites contain more than one trichome with more than 93 percent clusters containing more than two trichomes. Similar, although somewhat weaker, effects were found when introducing the cell-autonomously acting ICK1/KRP1^im^ construct into *cpc-try* plants with 39 percent clusters and 18 percent cluster with only two trichomes (Table 1). This shows that a reduction in endoreplication can override the effect of patterning mutants.

**Table 1:** Trichome initiation sites containing multiple trichomes on *try-cpc-PRO_GL2:_ICK1/KRP1* plants.

Genotype	Cluster^1^			Twin			Single			n
try cpc	94.0	±	4.6	1.3	±	2.5	4.7	±	4.8	21
#1 KRP1^109^ in try cpc	19.6	±	15.4	12.0	±	12.1	68.5	±	22.5	20
#2 KRP1^109^ in try cpc	29.2	±	13.9	16.1	±	6.0	54.6	±	10.1	20
try cpc x KRP1^109^	28.7	±	11.9	15.0	±	7.0	56.2	±	12.5	30
try cpc x KRP1^IM^	39.2	±	7.7	18.0	±	6.6	42.8	±	8.6	16

**1** A cluster is defined here as a trichome initiation site with more than 2 trichomes.

Next we analyzed crosses of *D*, *DE*, and *PRO_GL2_:ICK1/KRP1^im^* with *gl3* mutants, in which the trichome activator complex is compromised [22], [43], [52]. This combination resulted in a synergistic effect with a dramatic reduction of trichomes on leaf blades (Figure 3B, 3D, 3F, and 3G). Interestingly, we found on these plants a number of rudimentary, aborting trichomes that appeared to be arrested in their development and that we could never observe on wild-type plants (Figure 3D, 3F, and 3G). Taken together, these findings corroborate the importance of endoreplication, and place endoreplication control in an early phase of the trichome patterning process.

### Aborting trichomes lose their fate and transdifferentiate

**Figure 4:**
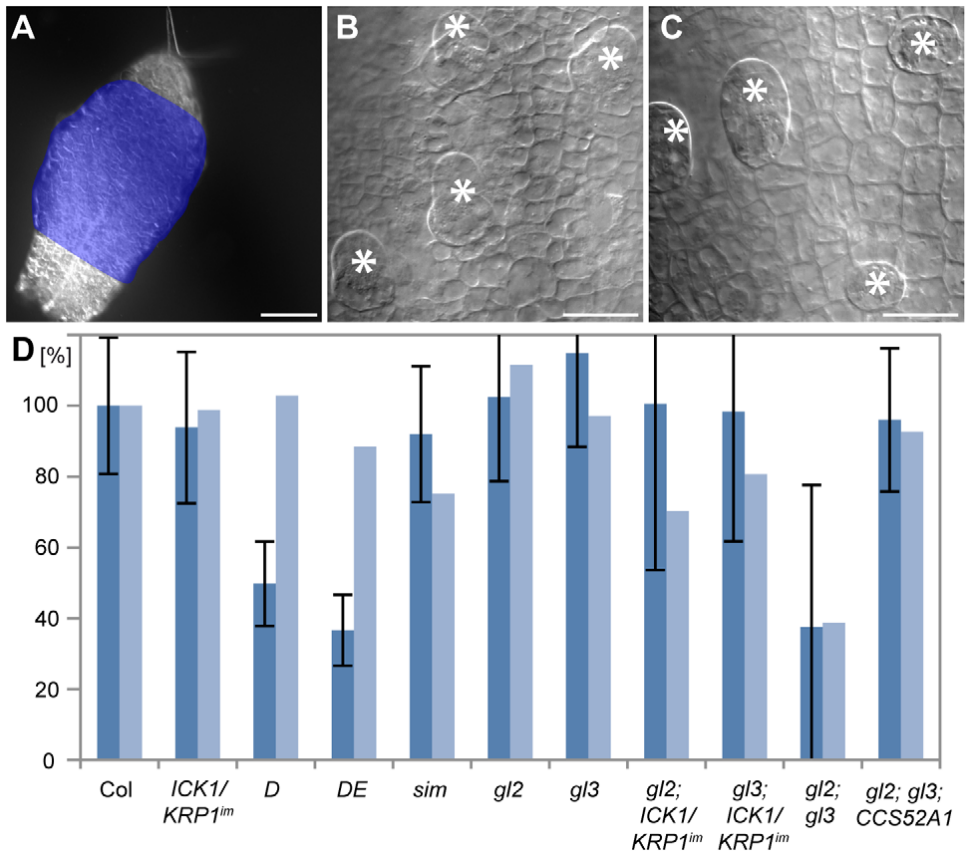
Analysis of early trichome initation. Light micrographs of young rosette leaves. (A) Overview of a young leaf 4 in wild type. The trichome initiation zone was defined as the leaf region where trichomes emerge (indicated in blue) and only unbranched trichomes were counted. (B) Trichome initiation zone in wild type (Columbia). (C) Trichome initiation zone in *CDKA;1^T161D^* leaves. (D) Quantification of the number of trichome initiation sites (TIS) compared to Columbia. Error bars: standard deviation n≥8. Scale bars: (A) 100 µm; (B,C) 25 µm.

The trichome pattern in Arabidopsis is established in the youngest part of a developing leaf. Therefore we analyzed the patterning zone of young leaves of plants with reduced endoreplication levels in trichomes. Consistent with previous studies [34], [41], [53], we found that in wild type emerging trichomes appeared with a minimal distance of approximately 3 cells (Figure 4A and 4B). As expected, epidermal cell size in the trichome patterning zone was much larger in *D* and *DE* plants than in wild type (Figure 4C). None-the-less, trichomes were patterned with roughly the same distance as in wild type resulting in a similar number of trichomes per cells (Figure 4C and 4D and Table S2). Thus, while the trichome pattern on old leaves is significantly different between wild-type plants and plants with a compromised endoreplication cycle, the initial pattern of trichomes appears to be rather similar.

**Figure 5:**
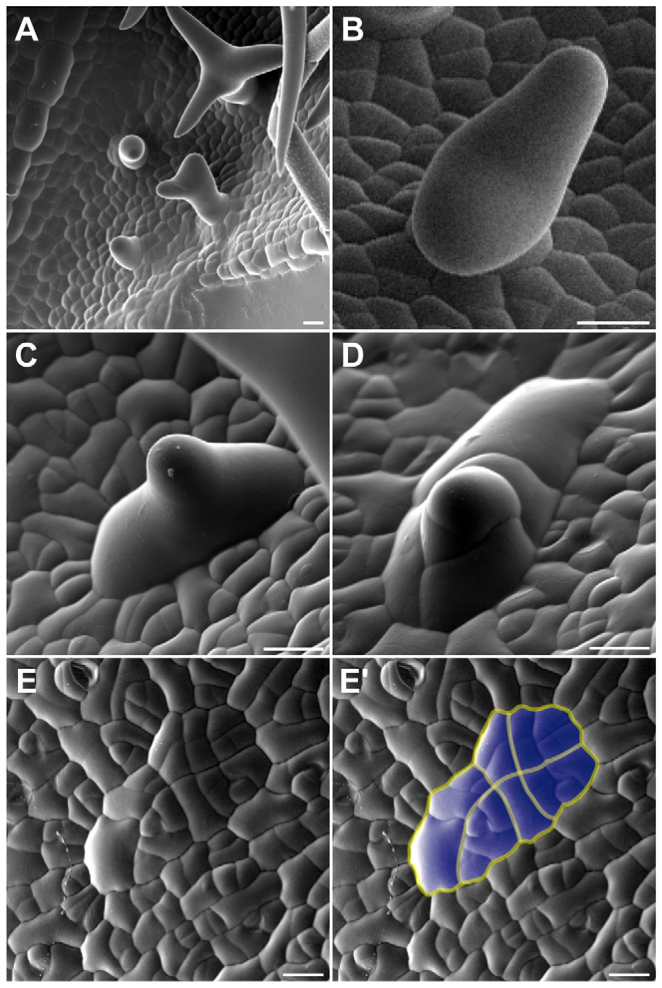
Morphology of aborted trichomes. Scanning electron micrographs of rosette leaves of wild-type plants (A,B) and plants expressing *PRO_GL2_:ICK1/KRP1* (C–E). (A) Trichome development on a young wild-type leaf. (B) Emerging wild-type trichome. (C) Aborting trichome on a young leaf of *PRO_GL2_:ICK1/KRP1* plant. (D) Putative aborted trichome undergoing cell division. (E) Possible remnants of an aborting trichome that underwent repeated rounds of cell division. Putative division planes indicated in (E′). Scale bars: (A) 30 µm; (B–D) 10 µm.

We therefore analyzed next how the trichome pattern becomes different over time in plants with reduced endoreplication levels and first examined in detail young leaves of these plants by scanning electron microscopy. We found that outside of the trichome patterning zone of leaves of *ICK1/KRP1*-mixexpressing plants as well as our other mutant lines several trichomes were arrested in their development, i.e. large cells with an outgrowth cone typical of developing trichomes, but with a much wider base (Figure 5C); these trichomes were never found on wild-type leaves (Figure 5A and 5B). Strikingly, we found a few cases where such an aborted trichome showed several constrictions suggesting recent cell divisions (Figure 5D). Finally, we found unusual patches of cells that displayed common division planes (Figure 5E). Typically, cell divisions in the wild-type leaf epidermis are not coordinated but mosaic whereas in the above identified cell patches division planes were aligned over more than eight cells (Figure 5E). One explanation for this common orientation could be that a large precursor cell would have undergone many successive divisions. However, the cell size of this precursor must have been very large, much larger than the typical epidermal pavement cell.

**Figure 6:**
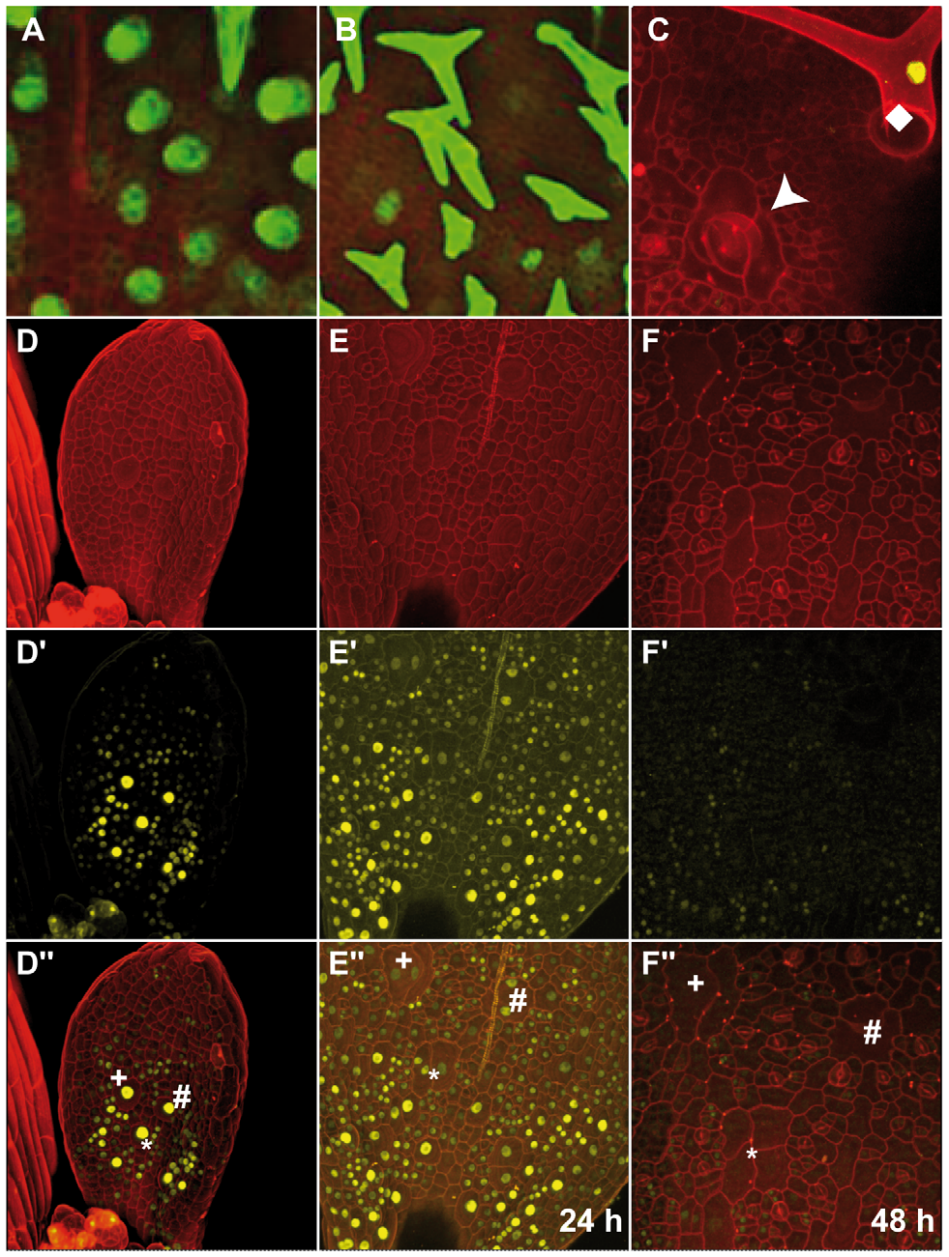
Live-imaging of aborting trichomes. Confocal-scanning micrographs of young rosette leaves; (A-C and D″-F″) overlay of propidium iodine channel and YFP/GFP channel; (D–F) Propidium iodine channel; (D′–F′) YFP/GFP channel. (A,B) Leaves of *PRO_GL2_:GFP* expressing plants in wild-type background after 24 h (A) and 48 h (B). (C) Leaf of *gl3*- *PRO_GL2_:GUS:YFP:ICK1/KRP1^109-191^* with a putative trichome precursor cell (arrow head) after cell division without YFP fluorescence while an outgrowing, i.e. non-aborting, trichome (square) displays a strong YFP signal. (D) Young leaf of *gl3*- *PRO_GL2_:GUS:YFP:ICK1/KRP1^109-191^*; young trichome cells can be identified by their increased size in comparison to the surrounding epidermal pavement cells and a bright fluorescent signal in the nucleus. Please note that some surrounding epidermal cells also display YFP fluorescence due to the low activity of the *GL2* promoter in groups of epidermal cells encompassing the future trichome. (E) The same leaf as in (D) after 24 h and after 48 h (F). Three trichome initials are marked by a plus, an asterisks, a number sign (D″–F″). Please note yellow background fluorescence in E′/E″ (cell walls are marked) and F′/F″ (some cell walls and stomata) as a consequence of the high sensitivity in the detection procedure.

To understand the origin of these cell patches and to test whether they could be derived from aborting trichomes, we followed *in vivo* the fate of trichome initials on very young leaves. We first monitored wild-type trichomes labeled by GFP expressed from the *GL2* promoter. We could track trichome initials developing into mature trichome cells during the time course of two days with pictures being taken every 24 hours; during the entire period the *GL2* reporter gave a strong fluorescence signal (Figure 6A and 6B). Since aborting trichomes are difficult to find, especially on young leaves that have a reduced number of epidermal cells in *D*, *DE* or *KRP*-misexpressing plants, we decided to follow trichome development in combinations of *ICK1/KRP1*-misexpressing plants with *gl3* mutants since these plants show one of the largest discrepancies between young and old leaves in terms of trichome numbers (compare Figure 3F and 3G with Figure 4D). In addition, we used for KRP expression an *ICK1/KRP1-YFP* fusion construct driven from the *GL2* promoter to mark trichomes and monitor at the same time the accumulation of the KRP protein.

Several leaves were observed over two to three days with pictures being taken every 24 hours. On many leaves we could observe that trichome initials were formed displaying strong YFP fluorescence similar to a fluorescent signal found in wild-type plants that express GFP under the control of the *GL2* promoter. In contrast to wild type, we could detect in a number of cases in which trichomes did not grow out further but underwent cell division. Figure 6 shows an example of a leaf in which three trichome initials divide, two initials undergoing one cell division giving rise to two cells and one initial dividing even twice leaving a patch of four enlarged cells that resembled the cluster that we had previously seen by SEM.

Moreover, in the aborting trichomes the YFP fluorescence rapidly diminished and was finally absent after two days. This could indicate that the KRP fusion protein would be rapidly degraded in this genetic background. However, non-aborting and outgrowing trichomes, as occasionally found on *gl3–PRO_GL2_: ICK1/KRP1-YFP* plants, showed a strong YFP fluorescence (Figure 6C). Thus, we conclude that in the aborting and dividing trichomes the *GL2* promoter is switched off indicating that along with the entry into a mitotic cell cycle these cells have lost their trichome identity.

To corroborate the trichome cell fate loss, we introgressed four trichome markers into *D* and *DE* plants, comprising of the 5′ regulatory region of *GL2*, *NOK*, the putative kinase *At2g36090* and the F-box protein F9C22.2 encoding gene *AT2G36090*, respectively [29], [54]. The *GL2* reporter was found to be expressed in young wild-type and aborting trichomes but, while its expression continued in wild-type trichomes, the activity of the reporter ceased in aborting trichomes (Figure S1). In contrast, the reporter construct for *NOK*, *At2g36090* and *F9C22.2* were only active in mature, three-branched trichomes and were not found to be expressed in aborting trichomes of mutants with reduced endoreplication levels (Figure S2, data not shown).

**Figure 7:**
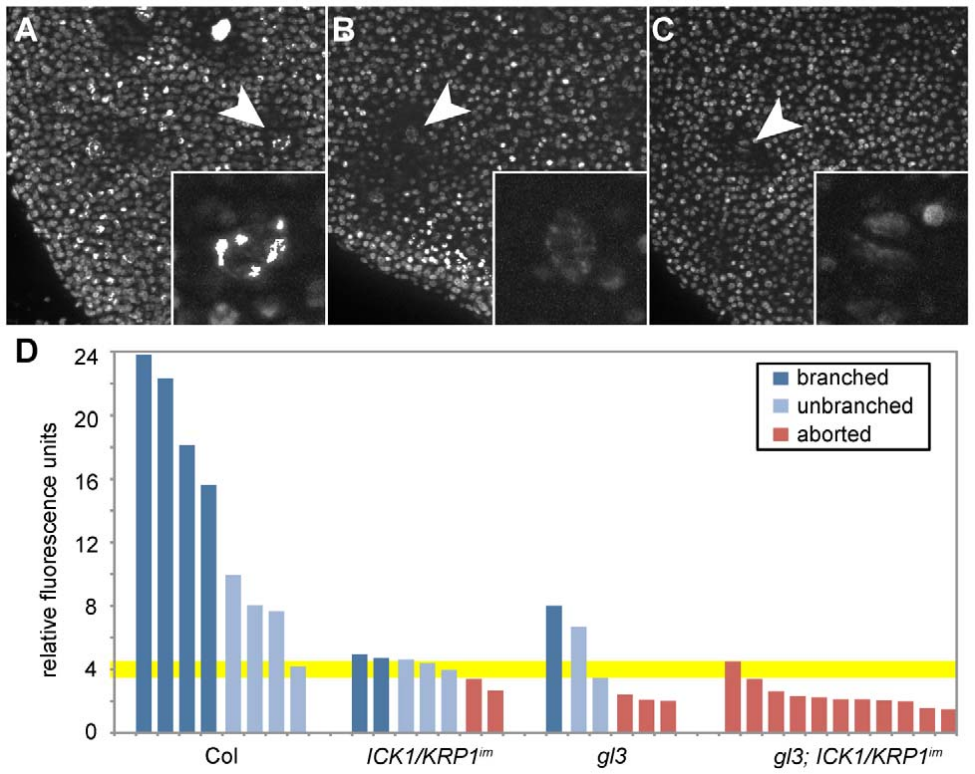
DNA content of young aborting trichomes. Light micrographs of young rosette leaves stained with DAPI. (A) Columbia wild-type plants. (B) *ICK1/KRP1^im^*. (C) *gl3 - ICK1/KRP1^im^*. (D) Quantification of DNA contents of trichome nuclei in relative fluorescence units (RFU). The RFU are calibrated by dividing epidermal cells nuclei so that 2 RFU roughly represents 2C.

Finally, we measured the DNA content of these aborting trichomes. The aborting trichomes were found to have large nuclei, sometime even larger than wild-type trichomes at comparable stages (see also Figure 6D′ and 6E′). However, nuclear size is well known to not inevitably reflect DNA content and indeed the fluorescence intensity of DAPI-stained trichome nuclei was much weaker of aborting trichomes than of wild type (Figure 7A–7C). Quantification of the exact DNA content of these aborting trichomes was difficult due to strong background fluorescent in young leaf parts and the often not clear distinction between a large epidermal cell and an aborting trichome during early developmental stages. None-the-less, our measurements always gave a similar trend with aborting trichomes having a DNA content between 2 and 4C, i.e. being not endoreplicated, in comparison with trichomes on wild-type plants at comparable stages that were found to have DNA levels between 4 and 8C (Figure 7D).

Taken together, our findings represent a case of trans-differentiation. Cells programmed to become trichomes and already expressing trichome identity genes changed their fate to an epidermal pavement cell program and were incorporated into the developing leaf epidermis.

### Promotion of endoreplication partially rescues aborting trichomes

**Figure 8:**
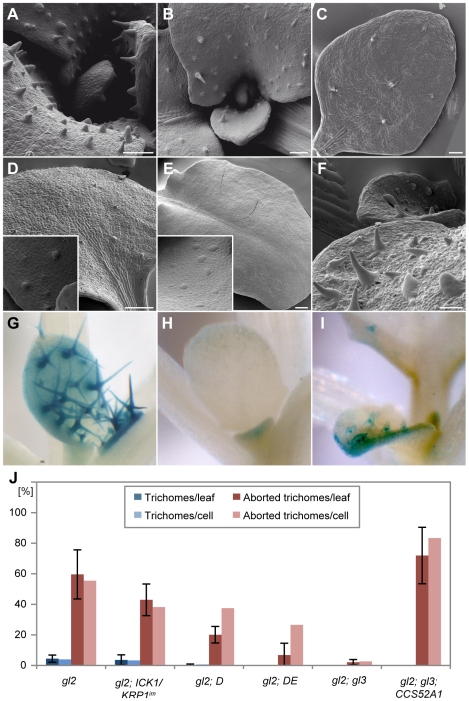
Partial rescue of trichome development of *gl2 gl3* by *PRO_GL2_:CCS52A1* expression. Scanning electron micrographs of rosette leaves (A–F) and light micrographs of GUS-stained leaves (G–I). (A) *gl2*. (B) *gl2 - ICK1/KRP1^im^*. (C) *gl2 - DE*. (D) *gl2 - sim*. (E) Mature leaves of *gl2–gl3* double mutant are completely devoid of trichomes. (F) *PRO_GL2_:CCS52A1* expression promotes endoreplication in trichome precursor cells and causes the formation of trichome-like cells in *gl2–gl3* double mutants. (G–I) Expression of the trichome marker *PRO_NOK_:GUS*. (G) Wild type. (H) In *gl2–gl3* mutants only a weak *PRO_NOK_:GUS* activity can be detected in leaf margins. (I) The trichome-like cells in *gl2–gl3-PRO_GL2_:CCS52A1* plants show *PRO_NOK_:GUS* activity. (J) Quantification of trichome number of leaf 3 and 4 in comparison with wild type.

Rudimentary trichomes also occur on leaves of *gl2* mutants and genetic combinations of *gl2* with *gl3* have a synergistic effect and were previously reported to display leaves completely devoid of trichomes [34], [55]. In the light of our above findings, we hypothesized that a decrease of ploidy levels in *gl2–gl3* double mutants might be the main factor responsible for the lack of trichomes. Therefore we introduced our set of lines with reduced endoreplication levels in trichomes into *gl2* mutants. These genetic combinations with *gl2* displayed a strong decline in trichome number and in the most severe cases (with the *gl2 DE* double mutant) all out-growing trichomes were eliminated (Figure 8A–8C and 8J and Table S1).

This raised the question whether trichomes were initiated in *gl2–gl3* double mutants but lose their fate similarly to the above made observations. Indeed, analysis by SEM revealed that large and distinct cells reminiscent of initial trichomes can be seen on young leaves but later on no traces of these cells are left (Figure 8E). We hypothesized that stimulation of a mitotic cycle in *gl2* mutants might further enhance trichome cell-fate loss. To test this we generated a *gl2-sim* double mutant and indeed the resulting double homozygous mutants resembled *gl2–gl3* mutants with no trichomes left on the leaf epidermis (Figure 8D). Again, we could find out-bulging cells on younger leaf parts indicating that trichomes were initially specified but lost their fate.

GL2 and GL3 are transcription factors that play a major role during trichome development and to test how important endocycle control versus a failure of regulating other target genes is, we sought for a way to specifically stimulate an endoreplication cycle and/or block mitosis in *gl2* and *gl2–gl3* mutants. Since it was recently shown that *CCS52* overexpression can induce endoreplication while blocking mitosis [56], [57], we generated a *PRO_GL2_:CCS52A1* construct and first introduced this into wild-type plants. Similar to the misexpression of *CCS52* from the *35S* promoter, its overexpressing under the *GL2* promoter control resulted in overbranched trichomes with an increase in trichome endoreplication levels but no obvious alteration of the trichome pattern was observed [56] (data not shown).

Next, we transformed the *PRO_GL2_:CCS52* construct into *gl2* and *gl2–g3* mutants. Indeed, the number of trichomes and trichome-like structures in *gl2* mutants that expressed *PRO_GL2_:CCS52* construct was higher than in *gl2* mutants (Figure S3A and S3B). The most striking effect was found in *gl2–gl3* double mutants expressing *CCS52*: While old leaves of *gl2–gl3* double mutants are devoid of trichomes, the *CCS52* overexpression lines displayed distinct cells with an outgrowth cone that resembled trichomes on *gl2* mutants (Figure 8F and 8J). These trichome-like structures never branched or developed papillae typical for mature trichomes. However, given that GL2 and GL3 are two major regulators of trichome development, a complete restoration to wild-type like trichomes was not expected.

To test the differentiation status of these cells, we introduced the above-used *NOK*, *At2g36090* and *F-box protein F9C22.2* promoter GUS reporter line into *gl2*, *gl3*, and into *gl2-gl3* mutants that expressed *CCS52* under the *GL2* promoter control. In all genotypes analyzed, the *At2g36090* and *F9C22.2* reporter were only active in outgrowing and neither in aborting trichomes nor in the trichome-like structures found in *gl2* or *gl2–gl3* mutants expressing CCS52 (Figure S2, data not shown). In contrast, the *NOK* reporter was expressed in outgrowing *gl2* mutant trichomes but was neither active in aborting trichomes in *gl2* nor in *gl2–gl3* mutants. However, in *gl2–gl3* mutants expressing *PRO_GL2_:CCS52* we observed that almost all rudimentary trichomes displayed a strong and enduring expression of this marker line (Figure 8G–8I). Similarly, *gl2* mutants expressing *PRO_GL2_:CCS52* construct showed activity of the NOK reporter in the partially restored trichomes that developed in the center of the leaves (Figure S3C and S3D). Thus, the block of the re-initiation of a mitotic program and promotion of endoreplication in *gl2* and in *gl2–gl3* double mutants is sufficient to at least partially maintain and promote trichome fate.

## Discussion

**Figure 9:**
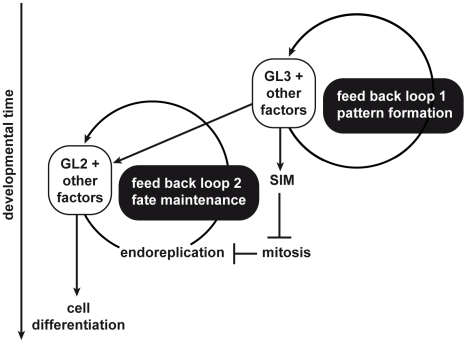
New model of trichome development. Trichome development appears to rely on two positive feed back loops. The first loop centers around the transcriptional activator GL3. The loop achieves the initial trichome pattern. Among other targets, GL3 directly activates *GL2* and *SIM* that are involved in a second feed back loop that is important for trichome cell fate maintenance and entry into an endoreplication cycle. Please note that in both feedback loops, GL3 and GL2 are operating together with many more regulators, indicated here as other factors.

Traditionally, trichome development has been divided into three separate phases, *pattern formation* from a field of initially equivalent epidermal cells. Next, *morphogenesis* with outgrowth and branch formation of the incipient trichome cell, accompanied by endoreplication. Finally, *maturation* with expansion growth and the formation of papillae on the surface [34]. Here we show that at least the two earlier phases have a substantial overlap with a new role for endoreplication and the repression of mitosis in maintaining and/or reinforcing the patterning process (Figure 9). Thus, in addition to a feedback loop of the trichome activator complex, including *GL3*, we postulate a second partially overlapping module with another feedback loop containing *GL2* acting during very early trichome pattern formation. This model is supported by the similarity of the *gl2–gl3* with the *gl2-sim* double mutant. In both cases trichomes abort shortly after their initial formation but–as demonstrated for *gl2–gl3*–can be rescued by promoting endoreplication and the inhibition of cell division. Moreover, the need for such second feedback loop during pattern formation gives a glimpse at the dynamics and complexity of tissue organization in living organisms.

### Endoreplication in trichome patterning

The data presented here show that endoreplication and the inhibition of mitosis are required for stabilization or maintenance of trichome cell fate, suggesting that there are developmental constraints on the patterning system. The switch from initial patterning to maintenance might be a sensitive phase in cell fate commitment, involving the transition from one major transcriptional program to another major transcriptional program, i.e. from a generic pattern module, also used for root hair patterning, to a cell type-specific readout, i.e. trichome morphology and physiology. Consistent with this view, genome-wide transcript profiling and the analysis of promoter reporter constructs have revealed that the expression of many patterning genes is strongly reduced in maturing trichomes [29] (and reference list there in). At the same time, these profiling studies have shown that *GL2* and *SIM* were among the most strongly expressed genes in mature trichomes in comparison to leaves without trichomes (99.4 and 28.0 fold, respectively). Since *GL2* was identified as a direct target of a patterning gene complex [58]–[60] (see also Figure 9), one needs to postulate a second transcriptional input after/or in parallel to the initial pattern formation. Similarly, *SIM* expression was found to positively correlate with *GL3* expression and chromatin immunoprecipitation experiments revealed that GL3 directly binds to the *SIM* promoter region [29], [35], [43]. It seems possible that endoreplication might be important to bridge these different transcriptional programs, for instance by increasing the intracellular concentrations of mRNAs for the proteins of the trichome activator complex (Figure 9).

Additionally, endoreplication is known to influence chromatin dynamics [61], and recent findings have even presented a molecular link between chromatin organization, pattern formation and cell-cycle control in particular during Arabidopsis root development. The Arabidopsis root epidermis is composed of alternating files of cells that all develop into hair cells (trichoblasts) or non-root hair cells (atrichoblasts). Root hair patterning and trichome patterning share many of the same regulators, such as *GL2* [21], [62]. Evidence that the chromatin state of *GL2* is connected to the specific cell fate in the root epidermis comes from the demonstration that the genomic region of *GL2* was only accessible to FISH hybridization in atrichoblasts where *GL2* is also expressed, and not in trichoblasts where the *GL2* promoter is not active [63]. Remarkably, the chromatin state was found to be very dynamic and could be established during a single cell cycle, likely between M-phase and G1 phase. It is plausible that endoreplication might influence chromatin accessibility by fixing a certain chromatin state in trichomes.

*GL2* expression was recently found to be also controlled by protein called GEM for GL2-EXPRESSION MODULATOR and *GEM* misexpression resulted in reduced levels of *GL2* whereas *gem* mutants displayed higher amounts of *GL2* mRNA [64]. Consistently, *gem* mutants produced more trichomes and *GEM* misexpression lines produced fewer trichomes. GEM was found to influence histone modifications, i.e. acetylation and methylation, around genes involved in trichome patterning, i.e. *GL2* and *CPC*, presenting one possibility for the expression control of *GL2*. GEM was found to interact with CDT1, a central component of the DNA replication machinery. CDT1 misexpression in turn enhances endoreplication levels in trichomes and trichome branch numbers [65]. In addition, in these *CDT1* misexpression lines, *GL2* is also upregulated offering a second link between DNA replication and *GL2* expression [64]. Thus, chromatin organization and DNA replication might be more intrinsically linked to pattern formation, and could represent the second feedback loop that we have postulated to function during early trichome patterning (Figure 9).

### Dedifferentiation and tissue patterning

The observation that trichomes can lose their fate and can be completely reintegrated into the pavement layer may shed light on general principles in pattern formation and tissue organization. A key question is which constraint underlies the necessity of a second feed back loop after pattern formation. Two major possibilities might explain this: The first scenario (cell-autonomous scenario) is based on the consideration that all epidermal cells, including trichome initials, are formed from an epidermal ground state. In this scenario, pavement cell fate would be the default state that must be overwritten during commitment of cells to the trichome fate. A failure to stabilize this program would reveal the default fate and accordingly, aborting trichomes would return to a pavement cell stage.

In this scenario, chromatin regulation might play a key role since it is known from Drosophila that cell fate is fixed and epigenetically inherited over many cell divisions by establishing repressive or activating chromatin states. Of key importance here are the Polycomb repressive complex one and two (PRC1 and PRC2) and the trithorax complex. The PRC2 mediates the tri-methylation of lysine 27 of Histone H3 leading to the recruitment of PRC1 complexes and the stable inactivation of the respective chromatin segment [66]–[68]. Homologs of the animal PRC2 complex have been identified in plants and among other target genes, *GL2* was found to be lysine K27 trimethylated in a PRC2-dependent manner [69] (D. Bouyer and A.S. unpublished data).

Another not mutually exclusively possibility is that the fate of an aborting trichome could be influenced by its neighboring cells (non-cell-autonomous scenario). Support for non-cell-autonomous influences on maintenance of cell fate come from grafting or ablation experiments where it was found that a cell or its progeny, when invading from one developmental context to another, adapts its fate according to its new position [70]–[73]. The existence of locally acting tissue-specific supervision mechanisms are presumably very important to organize and maintain body architecture by correcting incorrectly oriented cell divisions. However, mutants that affect the organization of tissue layers are very rare, likely due to the fundamental nature of this process and the probable pleiotropic mutant phenotype. One example might be mutants in the receptor-like kinase CR4 from maize or its Arabidopsis homolog ACR4 that have been found to be crucial for epidermis development. Both are expressed in the epidermal cell layer but may receive signals from underlying cell layers, thus also coordinating inter-tissue organization [74]–[78].

Trichome cell fate seems to be determined by a very robust developmental program since trichomes can be initiated and differentiate in subepidmermal layers in *try* mutants plants that ectopically express *GL1* [79]. It seems possible that endoreplication is one of the mechanisms that strongly stabilizes trichome fate and protects it from otherwise observed fate conversions induced by the neighboring cells. Interestingly, introgressing the patterning and endoreplication mutant *gl3* into *try-PRO_35S_:GL1* plants dramatically reduced the formation of subepidermal trichomes (A.S. and M.H., unpublished data). The emerging new tools to precisely study the development of single cell type for instance by laser dissection microscopy and a new round of mutant screens [31] will help to go in future one level deeper in the understanding of endoreplication during pattern formation and tissue integrity and will help to answer long standing questions in developmental biology.

## Materials and Methods

### Plant material and growth conditions

Arabidopsis (Arabidopsis thaliana) plants were grown on soil under long-day conditions (16 h of light, 8 h of darkness) between 18°C and 25°C at standard greenhouse conditions. To avoid the possibility of accession-specific variability in trichome development, only plants of the accession *Columbia-0* (*Col-0*) were used with the exception of the *cpc-try* double mutant, which is a combination of the accessions Landsberg *erecta* (*Ler*) and *Wasilewskaja-0* (*WS-0*) [44]. The *gl2*, *gl3* and *sim* mutants in *Col-0* have been described previously[29], [35], [80]. For a *cdka;1* mutant the previously characterized SALK T-DNA insertion allele was used [81]. The *CDKA; 1^T161D^* (*D*) and *CDKA; 1^T14D/Y15E^* (*DE*) rescue lines of *cdka;1−/−* were generated by Dissmeyer et al. (2007, 2009). The PRO_GL2_:*ICK1/KRP1*, PRO_GL2_:*ICK1/KRP1^109–191^* and *PRO_GL2_:GUS:YFP:KRP1^109–191^* (ICK/KRP1^im^) misexpression lines are characterized in Weinl et al. and Jakoby et al. [46], [49]. The PROMOTER-GUS reporter lines for *MYB106 At3g01140* and *At2g36090* are described in Jakoby et al. [51]. Genotypes were confirmed by PCR, antibiotic selection and/or segregation analysis of the following generation.

### Transgenic lines generated in this study

The *PRO_GL2_:ICK1/KRP1^109–191^* construct was transformed into *cpc-try* double mutants. For construction of *PRO_CPC_:CYCD3;1*, *PRO_TRY_:CYCD3;1*, and *PRO_GL2_:CCS52A1* the *Gateway* cloning system was used. The destination vectors *pAMPAT-PRO_CPC_* and *pAMPAT-PRO_TRY_* are a kind gift of Martin Pesch, University of Cologne [44], the *pAMPAT-PRO_GL2_* vector has been previously described [46]. Molecular manipulations were performed according to standard procedures and plants were transformed by a modified version of the floral dip method according to Clough and Bent [82]. At least 20 transgenic plants were generated for all expression constructs. A number of representative reference lines displaying a typical phenotype were chosen for further analysis. Genotypes were confirmed by PCR, antibiotic selection and/or segregation analysis of the following generation.

### Accession numbers

Sequence data for material used in this work can be found at TAIR (www.arabidopsis.org) and NCBI (www.ncbi.nlm.nih.gov) under the following accession numbers. For TAIR: At2g36090, At3g48750 (At CDKA;1), At2g46410 (At CPC), At4g34160 (At CYCD3;1), At2g36090 (At F9C22.2), At4g22910 (At FZR2/CCS52A1), At1g79840 (At GL2), At5g41315 (At GL3), At2g23430 (At ICK1/KRP1), At3g01140 (At NOK), At5g04470 (At SIM), At5g53200 (At TRY). Germplasm information for deposited T-DNA-lines: *cdka;1* (SALK_106809/Germplasm: 4824368), cpc (CS6399/Germplasm:1007963690), *gl3–3* (GK 545D05/Germplasm:3510637538), *sim-1* (Germplasm:5529955621), *try-EM1* (Germplasm:3510701804).

### Microscopy and image processing

Light microscopy was performed with an Axiophot microscope (Zeiss) and confocal laser scanning microscopy with a TCS SP2 AOBS CLSM system (Leica Microsystems). Scanning electron microscopy was done using a SUPRA 40VP (Zeiss) equipped with a K1250X Cryogenic SEM Preparation System (EMITECH). For image processing Leica Confocal Software Lite 2.05, Zeiss AxioVision 4.7, Adobe Photoshop CS2 and Adobe Illustrator CS2 were used. Image analysis was performed with Image J 1.43l (http://rsb.info.nih.gov/ij/).

### DNA measurements

For DNA quantification of trichome nuclei, rosette leaf 4 was vacuum infiltrated for 30 min in formaldehyde solution (3.7% formaldehyde in PBS, 0.1% Tween [PBST]) followed by incubation at 4°C overnight. Samples were washed two times for 15 min in PBST. Afterwards, leaves were vacuum infiltrated in DAPI solution (0.25 mg/mL, 5% DMSO in PBST) for 15 min and incubated overnight in DAPI solution at 4°C; thereafter, leaves were washed twice in PBST. The DAPI intensity was quantified and the background fluorescence was subtracted using the ImageJ software (rsbweb.nih.gov/ij/). The median value of *Columbia* and *gl3-3* trichomes was set as 32C and 16C respectively. From this value, the corresponding C values of the trichome nuclei were estimated. For a comparison of the DNA content of young trichomes and aborting trichomes in the initiation zone, Z-sacks of the initiation zone were taken with an ApoTome (Zeiss). The exact number and intensity of each pixel of the trichome nuclei were measured using the Z-axis profile plot function in ImageJ (rsbweb.nih.gov/ij/). The relative fluorescent units (RFU) were scaled by comparing to the RFU of trichome nuclei with nuclei of surrounding dividing epidermal cells. The smallest epidermal nuclei were set to 2C.

### Histology

The expression of the GUS protein was visualized as previously described [46]. For cell wall staining, leaves were directly mounted in a saturated solution of propidium iodide (100 µg/ml) in water and incubated for 5 min.

### Trichome counting

Trichomes were counted on leaf 3 and 4 at a leaf length of 4 mm length. Agarose (2% in Water) prints were taken of each leaf allowing an accurate measurement of the total leaf size as well as cell numbers and cell sizes by light microscopy. Total cell numbers and cell sizes per leaf were estimated by counting the number of cells in a square of 10000 µm^2^ located at ¼ and ¾ of the distance between tip and base of a leaf, halfway between midrib and leaf margin. All measurements were repeated three times on separately grown plants. For analyzing early pattering processes, trichome initiation sites (TIS) were counted in the trichome initiation zone of leaf 4 before it reached a length of 800 µm. The trichome initiation zone was defined at the most basal region of a leaf restricted at its distal end by appearance of branched trichomes [41] Total cell numbers and cell sizes in a trichome initiation zone were estimated by counting all pavement cells per square of 961 µm^2^. All measurements were repeated three times on separately grown plants.

### Trichome tracking

Plants were germinated and grown for 10 days on soil under long day conditions. Single leaves including the petioles and an upper part of the hypocotyls were cut off and put into a block of 1% MS agar so that the hypocotyls were embedded but the leaf blade was not in contact with the agar. These blocks were placed into Petri dishes and stored in a plant growth chamber under long day conditions for up to 72 hours. Each time before fluorescent images were obtained, leaves were stained in 200 µM propidium iodine for 5 min and washed with water. After image taking, the leaves were embedded to a new agar block and placed back into a Petri dish. Every 24 hours fluorescent images were taken by confocal laser-scanning microscopy using a 40x water-immersion objective without a cover slip.

## Supporting Information

Figure S1 Activity of *PRO_GL2_:GUS* in *DE* rosette leaves. Expression of *PRO_GL2_:GUS* in young (A) and mature trichomes (B). (C) GUS staining of an early aborting trichome (marked by an arrowhead). (D) Cell patch putatively derived from an aborted trichome with no GUS expression (marked by an arrow). Note the GUS positive surrounding young trichomes.
(0.91 MB TIF)

Figure S2 Expression of PRO_NOK_:GUS and PRO _At2g36090_:GUS. (A,C,E,G,I) PRO _At2g36090_:GUS activity in rosette leaves. (B,D,F,H,J) PRO_NOK_:GUS. (A,B) Wild-type Columbia. (C,D) *gl3*. E,F *gl2*. G,H *gl2-gl3*. I,J *gl2-gl3-PRO_GL2_:CCS52A1.*
(4.11 MB TIF)

Figure S3 Stereo-micrographs of rosette leaves (A,B) and light micrographs of GUS-stained rosette leaves (C,D). (A) *gl2* mutants predominantly form under-branched and small trichomes at the margin of leaves. (B) *gl2 - PRO_GL2_:CCS52A1* plants develop trichome-like structures on central leaf areas; one is indicated by an arrow. (C) *PRO_NOK_:GUS* activity can only be detected in *gl2* mutants in the outgrowing trichomes near leaf margins. (D) *PRO_NOK_:GUS* marks trichome like structures on *gl2 - PRO_GL2_:CCS52A1* plants. Scale bars: (A,B) 500 µm; (C,D) 100 µm.
(1.68 MB TIF)

Table S1 Trichome numbers and epidermal cell sizes of mature rosette leaves.
(0.02 MB XLS)

Table S2 Numbers and cell sizes of incipient trichomes and surrounding epidermal cells on young rosette leaves.
(0.02 MB XLS)
