# Supplementary figures and images for: Endoreplication Controls Cell Fate Maintenance
Source: PLoS Genet. 2010 Jun 24;6(6):e1000996. doi: 10.1371/journal.pgen.1000996 (PMC2891705; doi:10.1371/journal.pgen.1000996)

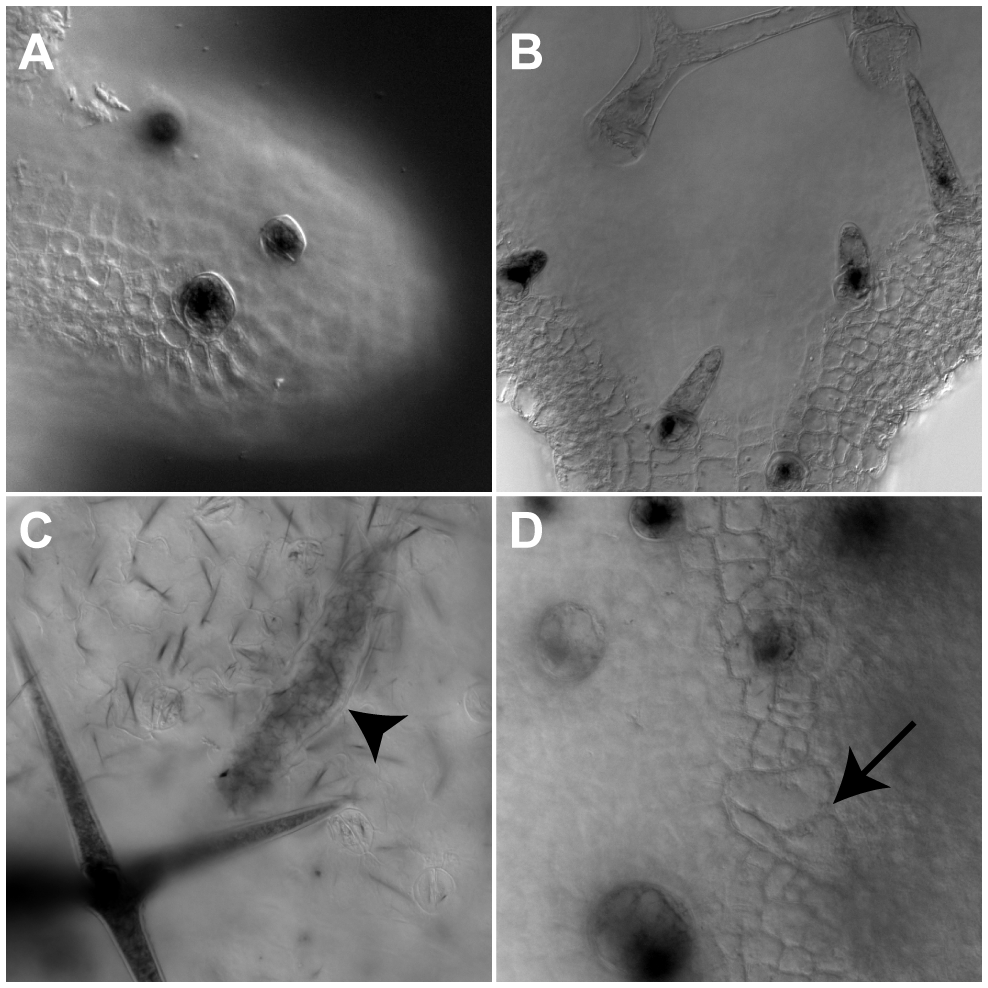

Supplement: Figure S1 — Activity of PROGL2:GUS in DE rosette leaves. Expression of PROGL2:GUS in young (A) and mature trichomes (B). (C) GUS staining of an early aborting trichome (marked by an arrowhead). (D) Cell patch putatively derived from an aborted trichome with no GUS expression (marked by an arrow). Note the GUS positive surrounding young trichomes. (0.91 MB TIF) [file pgen.1000996.s001.tif]

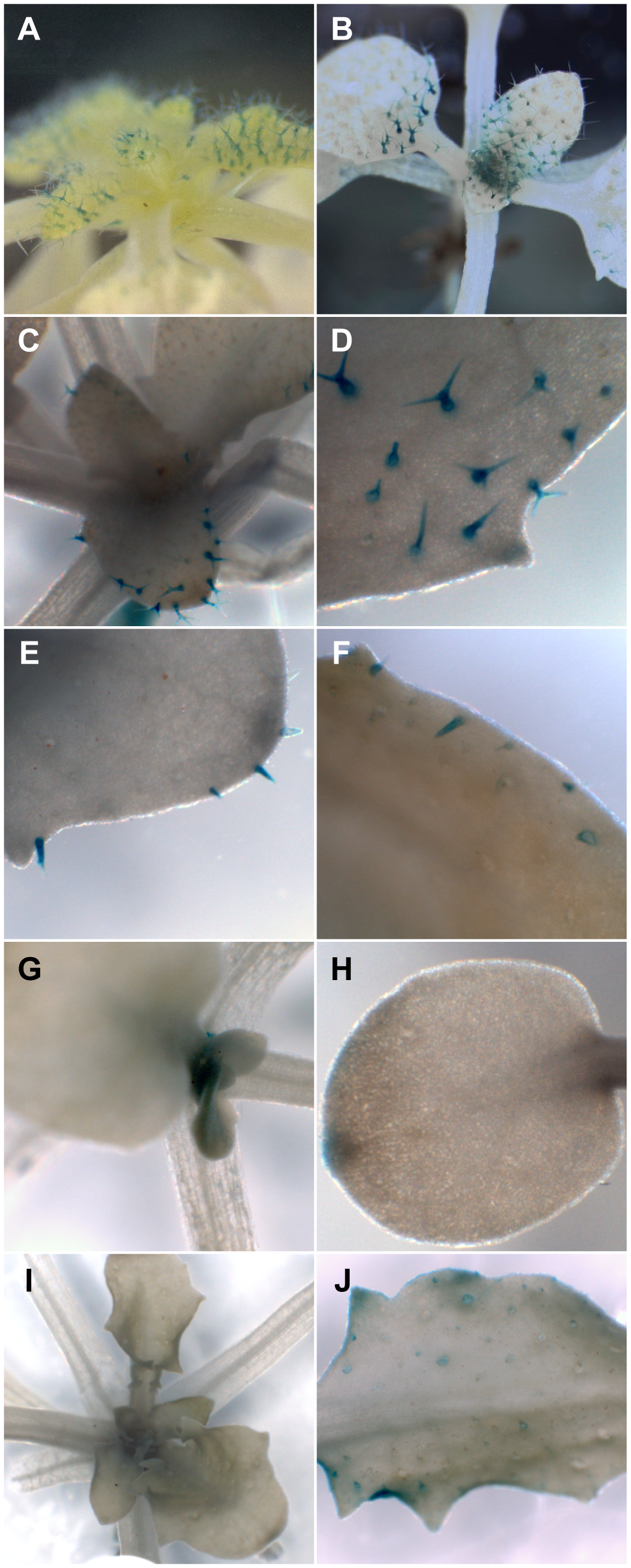

Supplement: Figure S2 — Expression of PRONOK:GUS and PRO At2g36090:GUS. (A,C,E,G,I) PRO At2g36090:GUS activity in rosette leaves. (B,D,F,H,J) PRONOK:GUS. (A,B) Wild-type Columbia. (C,D) gl3. E,F gl2. G,H gl2-gl3. I,J gl2-gl3-PROGL2:CCS52A1. (4.11 MB TIF) [file pgen.1000996.s002.tif]

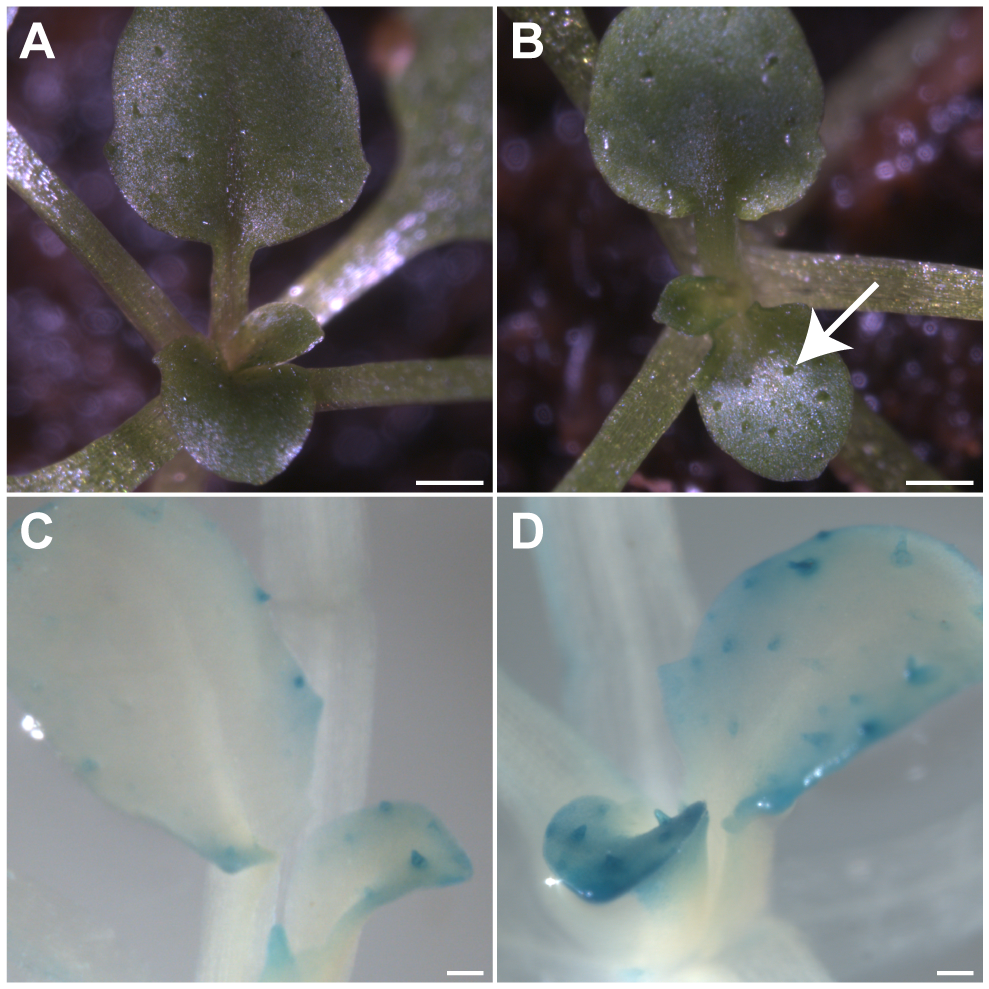

Supplement: Figure S3 — Stereo-micrographs of rosette leaves (A,B) and light micrographs of GUS-stained rosette leaves (C,D). (A) gl2 mutants predominantly form under-branched and small trichomes at the margin of leaves. (B) gl2 - PROGL2:CCS52A1 plants develop trichome-like structures on central leaf areas; one is indicated by an arrow. (C) PRONOK:GUS activity can only be detected in gl2 mutants in the outgrowing trichomes near leaf margins. (D) PRONOK:GUS marks trichome like structures on gl2 - PROGL2:CCS52A1 plants. Scale bars: (A,B) 500 µm; (C,D) 100 µm. (1.68 MB TIF) [file pgen.1000996.s003.tif]
